# Dietary and supplemental vitamin D intake and pain interference in adults with chronic spine-related pain: a cross-sectional survey study

**DOI:** 10.3389/fnut.2026.1815557

**Published:** 2026-05-07

**Authors:** Tao Chen, Yong Chen, Qiang Li, Li Su, Deng Zhao, Zhengjun Hu, Wenjing Zhang, Mengqiu Liang, Xue Lei, Juan Tan, Yinxiao Peng, Qiang Fu

**Affiliations:** 1 Department of Anesthesiology, The Third People's Hospital of Chengdu, Chengdu, China; 2 Department of Anesthesiology, Sichuan Provincial Orthopedic Hospital, Chengdu, China; 3 Department of Orthopedics, The Second Affiliated Hospital of Chongqing Medical University, Chongqing, China; 4 Department of Orthopedics, Chengdu BOE Hospital, Chengdu, China; 5 Health Management Center, The Third People's Hospital of Chengdu, Chengdu, China; 6 Department of Orthopedics, The Third People's Hospital of Chengdu, Chengdu, China

**Keywords:** body mass index, chronic spine-related pain, dietary intake, dietary supplements, pain interference, physical activity, vitamin D

## Abstract

**Background/objectives:**

Chronic spine-related pain is a leading driver of global disability, yet the role of modifiable nutritional factors in relation to functional impairment remains inadequately characterized. This study evaluated the associations between dietary vitamin D exposure ranking, vitamin D supplementation, and pain-related functional interference among adults in tertiary care settings.

**Methods:**

A multi-center cross-sectional survey was conducted at four tertiary hospitals in China, including 698 adults with spine-related pain persisting for at least 3 months. Dietary vitamin D intake was operationalized as a standardized ranking index derived from a semi-quantitative food frequency screener. Vitamin D supplementation was categorized by current and regular use. The primary outcome was the Brief Pain Inventory (BPI) interference mean score. Given the observational cross-sectional design, analyses were interpreted as associations rather than causal effects. Multivariable linear regression adjusted for confounders including age, BMI, physical activity, sleep quality, depressive symptoms, and sun exposure proxies.

**Results:**

Higher dietary vitamin D index was significantly associated with lower pain interference in the fully adjusted model (*β* = −0.18 per 1 SD increase; 95% CI: −0.29 to −0.07; *p* = 0.002). Regular vitamin D supplementation was also independently associated with reduced interference (*β* = −0.31; 95% CI: −0.52 to −0.10; *p* = 0.004). Associations were stronger for functional interference than for pain severity. Prespecified subgroup analyses revealed more pronounced dietary associations among participants with BMI ≥ 24 kg/m^2^ (*p*_interaction_ = 0.041) and those with low physical activity (*p*_interaction_ = 0.021).

**Conclusion:**

Higher dietary vitamin D exposure ranking and regular supplementation were independently associated with lower functional interference in this chronic spine pain population. Because the study was observational and cross-sectional, the findings should be interpreted as hypothesis-generating associations rather than evidence of therapeutic benefit, particularly in higher-risk phenotypes.

## 1 Introduction

Chronic spine-related pain is a dominant driver of disability and health service utilization, with low back pain consistently ranked as the leading cause of years lived with disability worldwide (1–3). In tertiary anesthesiology pain clinics and spine surgery pathways, the clinical problem extends beyond pain intensity: many patients experience substantial impairment in daily activity, walking, work, sleep, and enjoyment of life (4). This functional impact is strongly aligned with how patients judge recovery and how clinicians decide on escalation of care, including interventional procedures and pharmacologic strategies. The persistence of high symptom burden despite standard care has intensified interest in modifiable, scalable factors that may be associated with pain-related disability in real-world spine and pain clinic populations (5).

Vitamin D has emerged as one candidate factor because of its roles in musculoskeletal biology, neuromuscular function, and immune regulation (6). Vitamin D deficiency has been proposed as one factor potentially linked to musculoskeletal pain phenotypes through multiple pathways, including impaired muscle performance, altered inflammatory signaling, and downstream effects on fatigue, mood, and sleep, all of which may be relevant to pain-related disability (7, 8). At the same time, interpretation is complicated by nonrandom distribution of vitamin D exposure and status. Sun exposure, physical activity, adiposity, depressive symptoms, and sleep disturbance are intertwined with both vitamin D status and pain outcomes, creating substantial confounding risk in observational research and heterogeneity across clinical cohorts (9, 10).

The observational literature linking vitamin D status with pain has grown over the last decade, but conclusions remain mixed. A broad systematic review and meta-analysis of observational studies reported that lower circulating 25-hydroxyvitamin D concentrations were commonly associated with pain-related conditions, while also highlighting substantial heterogeneity across study designs and pain phenotypes (11). For low back pain specifically, systematic review evidence has supported an association between lower vitamin D levels and low back pain measures, but the magnitude and consistency of findings vary and are sensitive to study setting, phenotype definition, and covariate control (12). More recent work continues to report associations between vitamin D deficiency and chronic musculoskeletal pain outcomes, but also emphasizes inconsistency and the likelihood of residual confounding (13, 14). A practical limitation is that many prior studies prioritize pain intensity and serum measures, whereas disability-oriented outcomes and clinic-relevant patient-reported interference measures are less often positioned as the primary endpoint (15).

Randomized trial evidence has not fully resolved the question. A quantitative meta-analysis of randomized controlled trials evaluating vitamin D supplementation for pain reported conflicting results across conditions and trial designs, suggesting that any benefit may be modest and context-dependent (15, 16). In chronic low back pain, a recent meta-analysis focusing on randomized trials concluded that efficacy remains debated, with limited overall benefit across included studies. Trial heterogeneity is likely driven by differences in baseline deficiency status, achieved vitamin D repletion, dose and duration, co-interventions, and the underlying pain phenotype (16). Such variability has encouraged a more targeted framing: rather than expecting uniform analgesia, vitamin D may have stronger relevance for functional interference in subgroups where neuromuscular performance, sleep, and mood contribute meaningfully to disability (17).

Causal-inference approaches have also been applied. Mendelian randomization studies and evidence syntheses have evaluated whether genetically predicted vitamin D status relates to multiple health outcomes, including pain-related conditions (17). A Mendelian randomization analysis focused on low back pain reported that genetically higher serum 25-hydroxyvitamin D levels were associated with reduced risk of low back pain in European ancestry datasets (18). Even when such analyses suggest causality, translation to clinic decision-making is not straightforward because Mendelian randomization estimates reflect lifelong genetic differences, not short-term changes from diet or supplementation in symptomatic patients, and may not capture phenotype-specific pathways driving disability in tertiary care cohorts (17).

Spine surgery and spinal deformity care add another layer of relevance. Vitamin D deficiency has been discussed as a potentially modifiable factor affecting spine surgery outcomes, including patient-reported disability measures, fusion biology, and recovery trajectories (19). A meta-analysis of elective spinal fusion studies reported worse postoperative patient-reported outcomes in vitamin D deficient patients, supporting the clinical plausibility of vitamin D as a factor linked to functional status in spine populations (20). Narrative reviews in spine surgery have similarly emphasized that low preoperative vitamin D may predispose to worse outcomes, while acknowledging that evidence remains incomplete and heterogeneous (19). Related reviews have summarized associations between vitamin D deficiency and outcomes after spinal surgery and have called for higher-quality studies to clarify the role of screening and repletion strategies (21). Collectively, this spine-focused literature reinforces that vitamin D is clinically salient in tertiary spine settings, but it has not yet been evaluated consistently using disability-oriented pain outcomes in multi-center outpatient cohorts managed by anesthesiology pain services and spine clinics.

A further gap concerns exposure definition. Serum 25-hydroxyvitamin D is informative but not always available in routine clinic workflows and may vary with season, sun exposure, and acute behavior changes (22, 23). Dietary intake and supplementation are actionable targets for counseling and perioperative optimization, but intake measurement faces real limitations due to heterogeneous fortification practices and variable product composition (23, 24). For this reason, an intake-ranking approach may be more defensible than attempting precise IU per day estimation from short intake screeners in multi-center settings (23).

Accordingly, the present multi-center cross-sectional study evaluated associations between dietary vitamin D exposure ranking and vitamin D supplementation with pain-related functional interference among adults with chronic spine-related pain recruited from anesthesiology pain clinics, spine surgery clinics, and rehabilitation clinics at four tertiary hospitals. Pain interference was defined as the primary outcome using the Brief Pain Inventory interference score, with pain severity as a secondary outcome (25). Based on biological plausibility and prior heterogeneous evidence, we hypothesized that higher dietary vitamin D exposure ranking and regular vitamin D supplementation would be associated with lower pain interference after adjustment for prespecified confounders including physical activity, sleep quality, depressive symptoms, sun exposure proxies, season, analgesic use, and pain characteristics (26). We also prespecified exploratory effect modification by BMI and physical activity because both factors influence vitamin D physiology and may reflect distinct disability-driving pain phenotypes (24).

This study contributes multi-center, clinic-anchored evidence linking modifiable vitamin D related exposures to a disability-oriented pain endpoint in a population directly relevant to anesthesiology pain management and spine care. By prioritizing pain interference, using validated instruments for pain, sleep, mood, and activity, and operationalizing dietary intake as an intake-ranking index suited to heterogeneous fortification environments, the work provides a pragmatic framework for risk stratification and hypothesis generation. If confirmed, the findings would support targeted prospective studies and pragmatic trials testing whether vitamin D intake optimization, alone or paired with rehabilitation and sleep-focused interventions, is associated with improved pain-related functional outcomes in chronic spine-related pain populations managed in tertiary centers.

## 2 Materials and methods

### 2.1 Study design and setting

A multi-center, cross-sectional questionnaire survey was conducted to evaluate associations between dietary and supplemental vitamin D exposure and pain-related functional interference among adults with chronic spine-related pain. Data were collected at four tertiary hospitals in China with established anesthesiology-led pain services and spine surgery outpatient clinics, including at least one high-volume center specializing in complex spinal deformity surgery. Participants were recruited from outpatient visits in anesthesiology pain clinics, spine surgery clinics, and rehabilitation clinics. Data were collected between June 1, 2025 through September 12, 2025. Because the study used a cross-sectional design, exposure and outcome data were collected at the same survey time point, and temporal ordering could not be established.

### 2.2 Participants

Adults were eligible if they were aged 18 years or older and reported spine-related pain (cervical, thoracic, or lumbar region) persisting for at least 3 months. Participants were excluded if they reported spine surgery within the preceding 3 months, cancer-related pain, or inability to complete a self-administered questionnaire due to severe cognitive impairment or other conditions precluding valid survey completion. Screening was embedded at the beginning of the questionnaire, and ineligible individuals exited the survey after receiving a standardized message.

### 2.3 Recruitment and survey administration

Consecutive sampling was used in each participating clinic to reduce selection bias. Trained researchers approached potentially eligible patients during routine outpatient visits, provided standardized study information, and invited participation. After electronic consent, participants completed the questionnaire via Wenjuanxing by scanning a QR code. The platform was configured to prevent submission with missing responses for key instrument items. No direct identifiers such as name, national ID number, or phone number were collected.

A brief pilot (cognitive debriefing) was conducted before full rollout with 30 patients sampled at two centers to confirm item comprehension, completion time, and missingness patterns. Minor layout adjustments were made to improve clarity and reduce skip errors, while preserving standard wording for validated instruments.

### 2.4 Measures

#### 2.4.1 Primary outcome

Pain interference was assessed using the Brief Pain Inventory Short Form (BPI-sf). Participants rated interference over the prior week on 0 to 10 numeric rating scales for general activity, walking ability, normal work, sleep, and enjoyment of life. The primary outcome was the mean interference score (BPI interference mean), calculated as the average of the five interference items, with higher scores indicating greater functional interference. If one interference item was missing, the mean was calculated from the remaining items. If two or more interference items were missing, the mean was set to missing for analysis.

#### 2.4.2 Secondary pain outcomes

Pain severity was measured with BPI items rating worst, least, average, and current pain over the prior week (0–10). A pain severity mean was calculated as the average of the four severity items using the same missingness rule as above. Spine pain characteristics included primary pain region (cervical, thoracic, lumbar) and pain duration category (3 to 6 months, 6 to 12 months, 1 to 3 years, more than 3 years). Self-reported clinician-diagnosed spine conditions were recorded using a structured checklist including degenerative changes, disc herniation, spinal stenosis, spinal deformity (including scoliosis or kyphosis), and post-surgical spine pain outside the acute postoperative period.

#### 2.4.3 Dietary vitamin D exposure

Dietary vitamin D exposure was assessed using a brief semi-quantitative food frequency screener focused on major vitamin D-contributing foods over the prior 3 months. The screener captured intake frequency and usual portion size for milk or fortified milk, yogurt, cheese, fatty fish, eggs, fortified cereals or fortified beverages, and cod liver oil or vitamin D-fortified fish oil. The screener was designed as a pragmatic intake-ranking instrument for outpatient use rather than a full dietary assessment tool intended to estimate precise vitamin D intake in IU/day. This approach was selected because product fortification and nutrient composition vary across brands and settings, particularly in multi-center real-world clinical environments (23, 24, 27–29).

The food groups and response structure were adapted from standard semi-quantitative food frequency questionnaire principles used in nutritional epidemiology, but the specific brief screener used here was operationalized for this study as a focused vitamin D exposure-ranking tool rather than as a previously fully validated Chinese vitamin D questionnaire. Prior to rollout, cognitive debriefing was conducted in 30 patients at two centers to assess comprehension, completion burden, and response consistency, and minor layout modifications were made to improve clarity. Because formal criterion validation against serum 25-hydroxyvitamin D or a comprehensive dietary reference instrument was not performed, the dietary index should be interpreted as a relative ranking measure and not a direct surrogate for physiological vitamin D status.

Frequency categories were converted to weekly equivalents (0, 0.5, 1.5, 3.5, and 7 times/week). Portion categories were mapped to prespecified serving-size midpoints appropriate for the study setting. For each food group, a weekly intake index was calculated as frequency equivalent multiplied by portion midpoint. The dietary vitamin D index was then computed as the sum of food-group indices and standardized within the analytic sample to produce a continuous ranking variable. This standardized index was intended to rank participants from lower to higher probable vitamin D-related dietary exposure rather than to quantify absolute intake. Dietary exposure was analyzed as both a continuous standardized index and quantiles derived from the observed sample distribution.

#### 2.4.4 Vitamin D supplementation exposure

Supplement use was assessed with a structured module capturing current use (yes or no), clinician recommendation or prescription (yes, no, not sure), dose category (less than 400 IU, 400 to 800 IU, 800 to 2000 IU, more than 2000 IU, not sure), intake frequency category (daily, 4 to 6 days per week, 1 to 3 days per week, less than weekly), duration category (less than 6 months, 6 to 12 months, more than 12 months), and concurrent calcium supplementation (yes, no, not sure). Primary supplement exposure variables included current use (binary) and regular use (defined as daily or 4 to 6 days per week among current users). Dose category and duration category were used in secondary and sensitivity analyses. Because supplement dose, frequency, and duration were self-reported over a 3-month recall period, some recall error and exposure misclassification were possible.

#### 2.4.5 Sleep quality

Sleep quality over the prior month was assessed using the Pittsburgh Sleep Quality Index (PSQI) self-rated items. The PSQI global score was calculated using the standard seven-component algorithm, yielding a total score ranging from 0 to 21, with higher scores indicating worse sleep. Poor sleep was defined using a conventional threshold of PSQI greater than 5 for secondary analyses.

#### 2.4.6 Physical activity

Physical activity was assessed using the International Physical Activity Questionnaire short form (IPAQ-SF) over the prior 7 days. Weekly metabolic equivalent minutes were calculated using standard coefficients (walking 3.3 METs, moderate activity 4.0 METs, vigorous activity 8.0 METs). A categorical activity level variable (low, moderate, high) was derived using standard IPAQ classification rules. Sitting time on weekdays (minutes per day) was recorded and used descriptively and in sensitivity analyses.

#### 2.4.7 Depressive symptoms

Depressive symptoms were assessed using the Patient Health Questionnaire-9 (PHQ-9) over the prior 2 weeks. The PHQ-9 total score was computed as the sum of nine items (range 0 to 27). Severity categories were defined using standard cut points, and clinically relevant depressive symptom burden was operationalized as PHQ-9 score of 10 or higher for secondary analyses. The PHQ-9 functional difficulty item was captured to characterize impairment.

#### 2.4.8 Sun exposure proxy and season

Vitamin D relevant sun exposure was approximated using self-reported average daily outdoor time category and regular sunscreen use (yes or no). Season at survey completion (spring, summer, autumn, winter) was recorded for confounding control.

#### 2.4.9 Analgesic use and health covariates

Analgesic exposure over the prior 7 days was recorded using a medication checklist including NSAIDs, acetaminophen, opioids, muscle relaxants, antidepressants used for pain, anticonvulsants used for pain, topical analgesics, and other agents with free-text specification. Use pattern was captured as mostly scheduled, mostly as-needed, both, or not applicable. Additional covariates included age, sex, height and weight (used to compute body mass index), education level, smoking status, alcohol use frequency, and comorbidity checklist including osteoporosis, diabetes, chronic kidney disease, chronic liver disease, thyroid or parathyroid disease, and clinician-diagnosed depression or anxiety.

### 2.5 Data management and quality control

Data were collected directly into Wenjuanxing and exported to a secure, access-controlled database for analysis. Automated range checks were applied during data entry for numeric fields (age, height, weight, time-to-sleep, sleep duration, activity minutes). A data dictionary with standardized variable names and coding was used across centers. Prior to analysis, data cleaning included checks for out-of-range values, inconsistent responses, and duplicate submissions.

### 2.6 Sample size

The primary analysis used multivariable linear regression with BPI interference mean as a continuous outcome. A target total sample of approximately 600 to 800 participants across centers was planned to provide adequate precision for small effect sizes after adjustment for key confounders and center indicators, while supporting prespecified subgroup and sensitivity analyses. Recruitment targets were set to promote representation across participating sites and clinic types.

### 2.7 Statistical analysis

Participant characteristics were summarized using mean and standard deviation or median and interquartile range for continuous variables and counts with percentages for categorical variables. Dietary vitamin D index and supplement exposure distributions were summarized overall and by site.

The primary association between vitamin D exposure and pain interference was evaluated using multivariable linear regression. Main exposure models included (1) dietary vitamin D index (standardized continuous and quantiles) and (2) supplement use variables (current use and regular use). Center was modeled using fixed indicators, and clinic type was included to account for differences in referral patterns and case mix. Core confounders selected *a priori* included age, sex, body mass index, education, physical activity, depressive symptoms, sleep quality, sun exposure proxy variables, season, smoking, alcohol use, pain region, and pain duration category. Collinearity was assessed using variance inflation factors, and model diagnostics included residual checks and influence assessment.

Effect modification analyses examined interaction terms for body mass index category, physical activity level, and depressive symptom burden, based on biological plausibility and clinical relevance. Sensitivity analyses included excluding participants reporting chronic kidney disease or chronic liver disease, excluding participants reporting post-surgical spine pain, and repeating models with alternate exposure operationalization (dietary index only, supplement use only, combined models). Missing data were handled using complete-case analysis for primary models. Multiple imputation by chained equations had been prespecified as a sensitivity approach if missingness for any key covariate exceeded 5%. Statistical significance was assessed using two-sided tests with an alpha of 0.05. Analyses were conducted using a standard statistical software environment with reproducible scripts. To examine whether the high prevalence of analgesic use materially influenced the primary association estimates, an additional sensitivity analysis further adjusted the fully adjusted interference model for major analgesic classes, including NSAIDs, acetaminophen, opioids, muscle relaxants, antidepressants used for pain, anticonvulsants used for pain, and topical analgesics.

### 2.8 Ethics and consent

Ethics approval was obtained from the Ethics Committee of The Third People’s Hospital of Chengdu (Approval number SY2432). Electronic informed consent was obtained from all participants before any questionnaire items were presented.

## 3 Results

**Figure 1:**
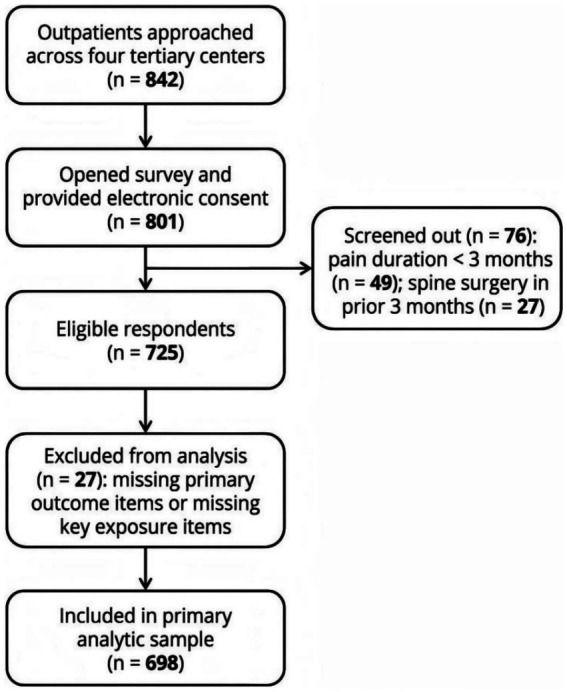
Participant flow diagram. Of 842 outpatients approached across four tertiary centers, 801 accessed the survey and provided electronic consent. Seventy-six were screened out because spine-related pain duration was under 3 months (*n* = 49) or spine surgery occurred within the prior 3 months (*n* = 27). Among 725 eligible respondents, 27 were excluded due to missing primary outcome items or missing key exposure items, leaving 698 participants in the primary analytic sample used for regression analyses of BPI pain interference and secondary pain severity outcomes.

Across four participating centers, 842 outpatients were approached during routine visits. Of 801 who accessed the survey and provided electronic consent, 76 were screened out because spine-related pain duration was under 3 months (*n* = 49) or spine surgery had occurred within the prior 3 months (*n* = 27). Among 725 eligible respondents, 27 submitted surveys missing primary outcome items for pain interference or missing key exposure items required to compute the dietary index. The final analytic sample included 698 participants. (Figure 1).

Item-level missingness was low for core instrument items because Wenjuanxing settings required completion for key scale questions. Because missingness for all key covariates remained below the prespecified 5% threshold, multiple imputation was not triggered, and complete-case analysis was retained as the primary approach. Covariate missingness ranged from 0.0 to 3.7%, highest for weight and weekday sitting time. Internal consistency in the analytic sample was high for BPI interference (Cronbach *α* = 0.90) and BPI severity (Cronbach *α* = 0.88), adequate for PSQI global scoring components (Cronbach *α* = 0.79), and high for PHQ-9 (Cronbach *α* = 0.86).

### 3.1 Baseline characteristics

Table 1 summarizes participant characteristics. Mean age was 47.8 years (SD 13.2) and 58.2% were female. Mean BMI was 24.7 kg/m^2^ (SD 3.8). Lumbar pain was the most common primary region (62.2%), followed by cervical (25.5%) and thoracic (12.3%). Pain duration exceeded 3 years in 41.1, and 33.4% reported pain for 1 to 3 years. Degenerative spine disease was the most frequently self-reported clinician diagnosis (54.9%), followed by disc herniation (36.7%), spinal stenosis (21.2%), post-surgical spine pain outside the acute period (18.2%), and spinal deformity including scoliosis or kyphosis (14.3%).

**Table 1:** Baseline characteristics of the analytic sample (*N* = 698).

Characteristic	Overall
Site	
Center A	210 (30.1%)
Center B	176 (25.2%)
Center C	162 (23.2%)
Center D	150 (21.5%)
Clinic type	
Anesthesiology pain clinic	322 (46.1%)
Spine surgery clinic	281 (40.3%)
Rehabilitation clinic	79 (11.3%)
Other	16 (2.3%)
Age, years	47.8 ± 13.2
Female	406 (58.2%)
Education	
Primary or below	78 (11.2%)
Secondary	232 (33.2%)
College or university	328 (47.0%)
Postgraduate	60 (8.6%)
Height, cm	165.2 ± 8.7
Weight, kg	67.3 ± 12.4
BMI, kg/m^2^	24.7 ± 3.8
Primary pain region	
Cervical	178 (25.5%)
Thoracic	86 (12.3%)
Lumbar	434 (62.2%)
Pain duration	
3 to 6 months	66 (9.5%)
6 to 12 months	112 (16.0%)
1 to 3 years	233 (33.4%)
More than 3 years	287 (41.1%)
Self-reported clinician diagnosis	
Degenerative changes	383 (54.9%)
Disc herniation	256 (36.7%)
Spinal stenosis	148 (21.2%)
Spinal deformity (scoliosis or kyphosis)	100 (14.3%)
Post-surgical spine pain (non-acute)	127 (18.2%)
Not sure	141 (20.2%)
BPI pain severity mean (0–10)	5.9 ± 1.8
Worst pain (0–10)	7.2 ± 1.7
Average pain (0–10)	5.8 ± 1.9
BPI pain interference mean (0–10)	5.6 ± 2.1
Interference with activity	5.9 ± 2.4
Interference with walking	5.5 ± 2.6
Interference with work	5.7 ± 2.5
Interference with sleep	6.0 ± 2.6
Interference with enjoyment of life	5.0 ± 2.5
PSQI total score (0–21)	9.2 ± 4.1
Poor sleep (PSQI > 5)	505 (72.3%)
PHQ-9 total score (0–27)	8 (4 to 12)
PHQ-9 ≥ 10	240 (34.4%)
Physical activity (IPAQ category)	
Low	284 (40.7%)
Moderate	296 (42.4%)
High	118 (16.9%)
IPAQ total MET-min/week	1,380 (660 to 2,460)
Sitting time, min/day (weekday)	420 (300 to 540)
Sun exposure and season	
Outdoor time < 15 min/day	148 (21.2%)
Outdoor time 15–30 min/day	214 (30.7%)
Outdoor time 30–60 min/day	220 (31.5%)
Outdoor time > 60 min/day	116 (16.6%)
Regular sunscreen use	318 (45.6%)
Spring	162 (23.2%)
Summer	186 (26.6%)
Autumn	176 (25.2%)
Winter	174 (24.9%)
Vitamin D supplementation	
Current vitamin D supplement use	266 (38.1%)
Regular use (daily or 4 to 6 days/week)	169 (24.2%)
Calcium co-supplementation	214 (30.7%)
Analgesic use in past 7 days	
Any analgesic use	602 (86.2%)
NSAIDs	418 (59.9%)
Acetaminophen	96 (13.8%)
Opioids	102 (14.6%)
Muscle relaxants	141 (20.2%)
Antidepressants for pain	76 (10.9%)
Anticonvulsants for pain	88 (12.6%)
Topical analgesics	204 (29.2%)
Mostly scheduled use pattern	248 (35.5%)
Lifestyle and comorbidities	
Current smoking	142 (20.3%)
Alcohol use ≥ 1 day/week	184 (26.4%)
Osteoporosis	92 (13.2%)
Diabetes	68 (9.7%)
Chronic kidney disease	18 (2.6%)
Chronic liver disease	16 (2.3%)
Thyroid or parathyroid disease	44 (6.3%)
Diagnosed depression or anxiety	98 (14.0%)

Values are mean ± SD, median (IQR), or n (%).

Pain burden was substantial. Mean BPI interference score was 5.6 (SD 2.1) and mean BPI severity score was 5.9 (SD 1.8). Sleep disturbance was common, with mean PSQI total 9.2 (SD 4.1) and 72.3% meeting the poor sleep threshold (PSQI > 5). Median PHQ-9 was 8 (IQR 4 to 12), and 34.4% had PHQ-9 ≥ 10. Current vitamin D supplement use was reported by 38.1% and regular use by 24.2%. Because recruitment occurred between June and September 2025, the measured dietary and behavioral exposure profile might reflect summer-to-early-autumn patterns and might not represent year-round average vitamin D-related exposure.

### 3.2 Dietary vitamin D index distribution and crude gradients in pain interference

The dietary vitamin D index, constructed as a semi-quantitative intake ranking measure and standardized in the analytic sample, showed an approximately symmetric distribution (mean 0.00, SD 1.00). Participants in higher dietary index quartiles were modestly older and less likely to report higher depressive symptom burden. Current supplement use was more frequent in higher dietary quartiles. Mean BPI interference declined across dietary index quartiles from 5.9 (SD 2.0) in Quartile 1 to 5.2 (SD 2.1) in Quartile 4 (*p* = 0.002), consistent with a graded relationship (Table 2).

**Table 2:** Characteristics by quartile of the dietary vitamin D index (*N* = 698).

Variable	Q1 Lowest (*n* = 174)	Q2 (*n* = 175)	Q3 (*n* = 175)	Q4 Highest (*n* = 174)	*p-*value
Age, years	46.1 ± 13.4	47.2 ± 13.1	48.3 ± 12.8	49.6 ± 13.2	0.041
Female	108 (62.1%)	106 (60.6%)	102 (58.3%)	90 (51.7%)	0.12
BMI, kg/m^2^	24.9 ± 3.9	24.8 ± 3.7	24.6 ± 3.8	24.4 ± 3.7	0.46
Lumbar pain region	112 (64.4%)	110 (62.9%)	109 (62.3%)	103 (59.2%)	0.72
Pain duration > 3 years	70 (40.2%)	68 (38.9%)	73 (41.7%)	76 (43.7%)	0.81
PSQI total	9.6 ± 4.2	9.4 ± 4.1	9.1 ± 4.0	8.6 ± 4.1	0.078
PHQ-9 total	9 (5 to 13)	8 (4 to 12)	8 (4 to 12)	7 (3 to 11)	0.031
Low activity (IPAQ)	78 (44.8%)	74 (42.3%)	71 (40.6%)	61 (35.1%)	0.18
Outdoor time > 60 min/day	22 (12.6%)	27 (15.4%)	31 (17.7%)	36 (20.7%)	0.11
Current vitamin D supplement use	56 (32.2%)	60 (34.3%)	72 (41.1%)	78 (44.8%)	0.014
Regular supplement use	35 (20.1%)	37 (21.1%)	46 (26.3%)	51 (29.3%)	0.048
BPI severity mean	6.1 ± 1.8	6.0 ± 1.8	5.8 ± 1.7	5.7 ± 1.8	0.045
BPI interference mean	5.9 ± 2.0	5.7 ± 2.1	5.5 ± 2.1	5.2 ± 2.1	0.002
Opioid use (past 7 days)	30 (17.2%)	28 (16.0%)	24 (13.7%)	20 (11.5%)	0.26

*p-*values from ANOVA, Kruskal-Wallis, or chi-square tests as appropriate.

### 3.3 Primary analysis: dietary vitamin D index and pain interference

**Figure 2:**
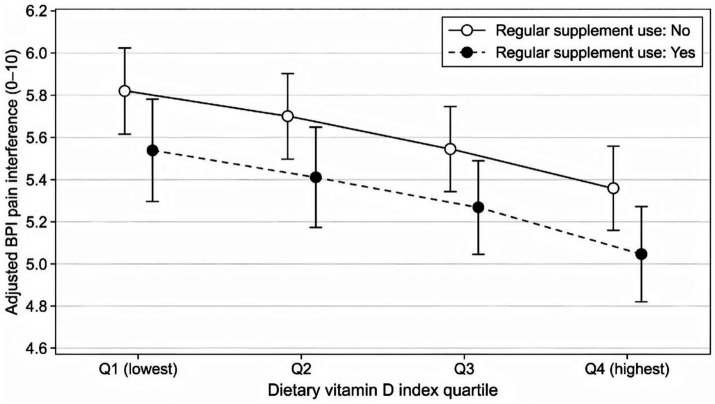
Adjusted mean pain interference across dietary vitamin D exposure quartiles, stratified by regular vitamin D supplementation. Adjusted marginal mean Brief Pain Inventory (BPI) pain interference score (0–10) across quartiles of the standardized dietary vitamin D exposure index, stratified by regular vitamin D supplementation (yes vs. no). Marginal means and 95% confidence intervals were obtained from the fully adjusted linear regression model including center, clinic type, age, sex, BMI, education, physical activity, depressive symptoms, sleep quality, outdoor time category, sunscreen use, season, smoking status, alcohol frequency, primary pain region, pain duration category, and analgesic use pattern. Higher dietary vitamin D exposure ranking was associated with lower adjusted pain interference, and regular supplementation was associated with a lower interference level across quartiles.

**Figure 3:**
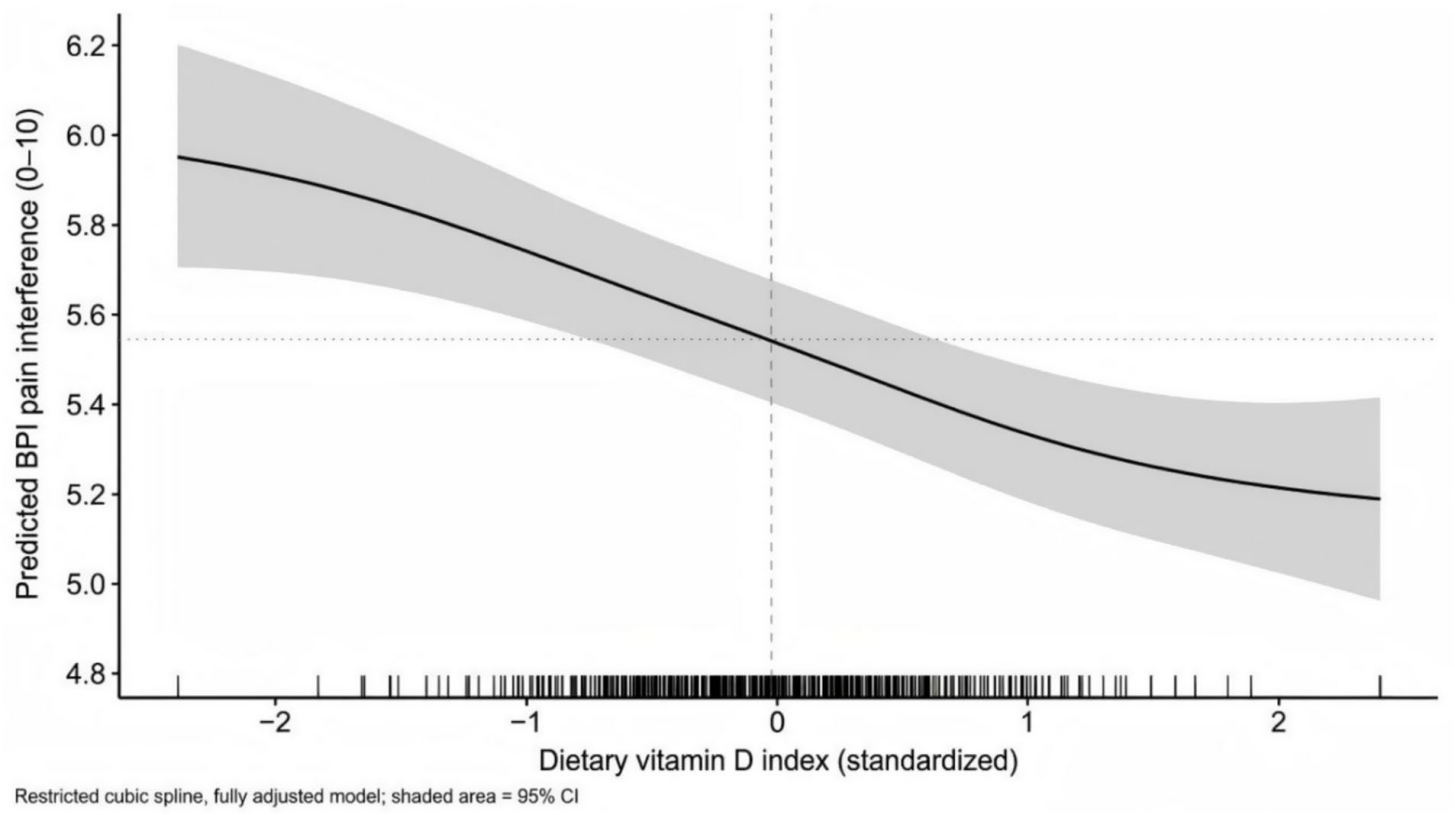
Restricted cubic spline of dietary vitamin D exposure and predicted pain interference. The association between the standardized dietary vitamin D exposure index and the predicted Brief Pain Inventory (BPI) pain interference score (0 to 10) from the fully adjusted linear regression model. The solid curve represents the adjusted predicted mean, and the shaded band represents the 95% confidence interval. The model adjusted for center, clinic type, age, sex, BMI, education, physical activity, depressive symptoms, sleep quality, outdoor time category, sunscreen use, season, smoking status, alcohol frequency, primary pain region, pain duration category, and analgesic use pattern.

In unadjusted linear regression, each 1 SD higher dietary vitamin D index was associated with 0.32 points lower BPI interference (*β* = −0.32; 95% CI -0.43 to −0.21; *p* < 0.001). The association remained after partial adjustment for center, clinic type, age, sex, BMI, and education (*β* = −0.27; 95% CI -0.38 to −0.16; *p* < 0.001). In the fully adjusted model incorporating physical activity, depressive symptoms, sleep quality, sun exposure proxies, season, smoking, alcohol use, pain region, pain duration, and analgesic use pattern, the estimate attenuated but remained statistically significant (*β* = −0.18; 95% CI –0.29 to −0.07; *p* = 0.002). The fully adjusted model explained a substantial proportion of outcome variance (*R*^2^ = 0.42), while the dietary index contributed a small but measurable independent association (Figures 2, 3).

### 3.4 Supplementation analysis and mutual adjustment with dietary index

Vitamin D supplementation showed similar directionality. In fully adjusted models, current supplement use was associated with lower pain interference (*β* = −0.22; 95% CI –0.40 to −0.04; *p* = 0.017). Regular supplement use had a larger association (*β* = −0.31; 95% CI –0.52 to −0.10; *p* = 0.004).

When dietary index and regular supplement use were modeled simultaneously with full adjustment, both remained independently associated with lower interference (dietary index *β* = −0.16; 95% CI -0.27 to −0.05; *p* = 0.005; regular supplement *β* = −0.28; 95% CI -0.49 to −0.07; *p* = 0.009), indicating that diet-related intake ranking and regular supplementation were not fully redundant exposures.

### 3.5 Secondary outcome: pain severity

Associations with BPI severity were directionally consistent but smaller. In fully adjusted models, the dietary index was associated with lower pain severity (*β* = −0.12; 95% CI -0.21 to −0.03; *p* = 0.010). Regular supplement use was also associated with lower severity (*β* = −0.19; 95% CI –0.36 to −0.02; *p* = 0.029). The magnitude difference between interference and severity outcomes was consistent with the study focus on functional impact.

The full regression results for interference and severity are shown in Table 3.

**Table 3:** Multivariable associations of dietary vitamin D index and supplementation with pain outcomes (*N* = 698).

Exposure	Model	BPI interference mean β (95% CI)	*p-*value	BPI severity mean β (95% CI)	*p-*value
Dietary vitamin D index (per 1 SD)	Model 0: Unadjusted	-0.32 (−0.43, −0.21)	<0.001	−0.19 (−0.28, −0.10)	<0.001
	Model 1: Partial adjustment	−0.27 (−0.38, −0.16)	<0.001	−0.15 (−0.24, −0.06)	0.001
	Model 2: Full adjustment	−0.18 (−0.29, −0.07)	0.002	−0.12 (−0.21, −0.03)	0.01
Current vitamin D supplement use (yes vs. no)	Model 2: Full adjustment	−0.22 (−0.40, −0.04)	0.017	−0.14 (−0.30, 0.02)	0.088
Regular supplement use (yes vs. no)	Model 2: Full adjustment	−0.31 (−0.52, −0.10)	0.004	−0.19 (−0.36, −0.02)	0.029
Dietary index + regular supplement (mutual adjustment)	Model 2: Full adjustment	Dietary: −0.16 (−0.27, −0.05)	0.005	Dietary: −0.10 (−0.19, −0.01)	0.027
		Regular: −0.28 (−0.49, −0.07)	0.009	Regular: −0.16 (−0.34, 0.02)	0.081

Estimates are linear regression β coefficients representing change in outcome points (0–10 scales). Because this was a cross-sectional observational study, *β* coefficients should be interpreted as adjusted associations and not as causal effects. Dietary vitamin D index is standardized, so *β* reflects change per 1 SD increase. Semi-partial *R*^2^ is shown for the exposure term in the fully adjusted model to summarize incremental variance explained by the exposure beyond covariates. Model definitions: Model 0: unadjusted. Model 1: center, clinic type, age, sex, BMI, education. Model 2: Model 1 plus physical activity (IPAQ), depressive symptoms (PHQ-9), sleep quality (PSQI), outdoor time category, sunscreen use, season, smoking status, alcohol frequency, pain region, pain duration category, analgesic use pattern. Model fit (*R*^2^): Interference outcome: Model 0 = 0.04; Model 1 = 0.18; Model 2 = 0.42. Severity outcome: Model 0 = 0.03; Model 1 = 0.12; Model 2 = 0.31. Incremental exposure contribution in Model 2 (semi-partial *R*^2^): Dietary index: 0.8% (interference), 0.5% (severity). Regular supplement use: 0.6% (interference), 0.3% (severity).

### 3.6 Prespecified effect modification and subgroup analyses

**Figure 4:**
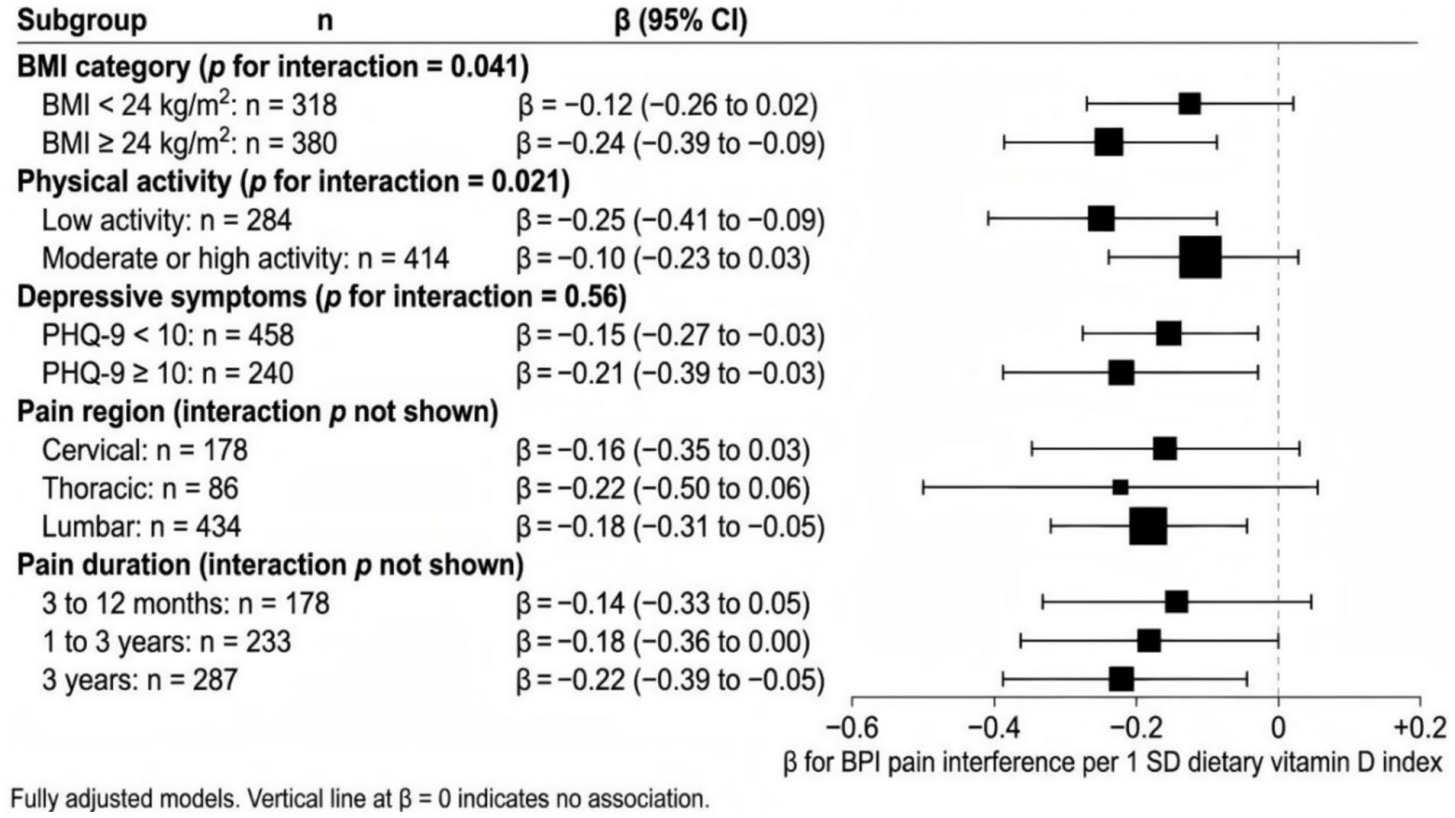
Subgroup associations between dietary vitamin D exposure and pain interference. Forest plot of fully adjusted associations between the standardized dietary vitamin D exposure index (per 1 SD increase) and Brief Pain Inventory (BPI) pain interference score across prespecified subgroups. Points indicate adjusted regression coefficients and horizontal lines indicate 95% confidence intervals. Models were adjusted for the full prespecified covariate set, including center, clinic type, age, sex, education, sleep quality, depressive symptoms, outdoor time category, sunscreen use, season, smoking status, alcohol frequency, primary pain region, pain duration category, and analgesic use pattern, with the relevant stratification variable omitted within each subgroup model. Interaction *p-*values are shown for BMI category, physical activity level, and depressive symptom burden.

Prespecified interaction analyses indicated heterogeneity in the dietary index association with pain interference by BMI category and physical activity. Among participants with BMI ≥ 24 kg/m^2^, the fully adjusted association was stronger (*β* = −0.24; 95% CI -0.39 to −0.09) than among those with BMI < 24 kg/m^2^ (β = −0.12; 95% CI -0.26 to 0.02), with interaction *p* = 0.041. Among participants classified as low activity, the association was *β* = −0.25 (95% CI -0.41 to −0.09), compared with β = −0.10 (95% CI -0.23 to 0.03) in moderate or high activity (interaction *p* = 0.021). No meaningful interaction was observed by depressive symptom burden (interaction *p* = 0.56). Full subgroup results are shown in Table 4 and Figure 4.

**Table 4:** Prespecified effect modification for dietary vitamin D index and pain interference (fully adjusted models).

Subgroup	*n*	*β* (95% CI)	*p-*value	*p* for interaction
BMI category				0.041
BMI < 24 kg/m^2^	318	−0.12 (−0.26, 0.02)	0.094	
BMI ≥ 24 kg/m^2^	380	−0.24 (−0.39, −0.09)	0.002	
Physical activity				0.021
Low activity	284	−0.25 (−0.41, −0.09)	0.002	
Moderate or high activity	414	−0.10 (−0.23, 0.03)	0.13	
Depressive symptoms				0.56
PHQ-9 < 10	458	−0.15 (−0.27, −0.03)	0.014	
PHQ-9 ≥ 10	240	−0.21 (−0.39, −0.03)	0.021	

Dietary vitamin D index is per 1 SD. Outcome is BPI interference mean. All subgroup models use the full adjustment set, excluding the stratification variable where appropriate.

### 3.7 Sensitivity analyses

Estimates were stable across sensitivity analyses (Table 5). Excluding participants reporting chronic kidney disease or chronic liver disease (analytic *n* = 664) yielded dietary index *β* = −0.17 (95% CI –0.29 to −0.05; *p* = 0.006) and regular supplement *β* = −0.27 (95% CI -0.49 to −0.05; *p* = 0.016). Excluding participants with post-surgical spine pain (analytic *n* = 571) produced dietary index *β* = −0.19 (95% CI − 0.32 to −0.06; *p* = 0.004) and regular supplement *β* = −0.30 (95% CI –0.55 to −0.05; *p* = 0.019). Replacing PSQI total with the binary poor sleep indicator did not materially change results. Complete-case analysis produced similar effect estimates.

**Table 5:** Sensitivity analyses for pain interference outcome (Model 2 specification).

Sensitivity analysis	*n*	Dietary index *β* (95% CI)	*p-*value	Regular supplement *β* (95% CI)	*p-*value
Primary Model 2	698	−0.18 (−0.29, −0.07)	0.002	−0.31 (−0.52, −0.10)	0.004
Excluding CKD or chronic liver disease	664	−0.17 (−0.29, −0.05)	0.006	−0.27 (−0.49, −0.05)	0.016
Excluding post-surgical spine pain	571	−0.19 (−0.32, −0.06)	0.004	−0.30 (−0.55, −0.05)	0.019
Replace PSQI total with poor sleep indicator	698	−0.17 (−0.28, −0.06)	0.003	−0.30 (−0.51, −0.09)	0.005
Complete-case for all covariates	666	−0.19 (−0.31, −0.07)	0.002	−0.33 (−0.55, −0.11)	0.003

CKD: chronic kidney disease. Dietary vitamin D index is per 1 SD. Regular supplement use is yes vs no.

Additional sensitivity analysis further adjusting the fully adjusted interference model for major analgesic classes yielded materially similar results, suggesting that the observed associations were not explained solely by broad differences in medication exposure. After additional adjustment for NSAIDs, acetaminophen, opioids, muscle relaxants, antidepressants used for pain, anticonvulsants used for pain, and topical analgesics, the dietary vitamin D index remained associated with lower pain interference (*β* = −0.16, 95% CI –0.27 to −0.05, *p* = 0.005) and regular supplement use remained associated with lower pain interference (*β* = −0.28, 95% CI –0.48 to −0.08, *p* = 0.006).

## 4 Discussion

In this multi-center cross-sectional outpatient sample of adults with chronic spine-related pain, higher dietary vitamin D exposure ranking and vitamin D supplementation were each associated with lower pain-related functional interference. The association for dietary exposure remained statistically significant after adjustment for a broad set of confounders spanning pain phenotype, physical activity, depressive symptoms, sleep quality, sun exposure proxies, season, analgesic use pattern, and sociodemographic factors. Supplement use showed a similar pattern, with regular use demonstrating a larger association than current use alone. When dietary exposure ranking and regular supplementation were modeled jointly, both retained independent associations with pain interference, suggesting partially distinct or additive correlates. Secondary analyses indicated smaller associations for pain severity than for pain interference, reinforcing the value of disability-oriented endpoints in spine pain research. Prespecified interaction analyses suggested stronger dietary associations among participants with higher BMI and among participants with low physical activity, while associations did not materially differ by depressive symptom burden. Sensitivity analyses excluding participants with chronic kidney or liver disease and excluding participants with post-surgical spine pain yielded similar results, supporting robustness to clinically relevant comorbidity and phenotype exclusions.

Observational evidence linking vitamin D to pain outcomes remains heterogeneous, and the present findings add a clinic-anchored perspective by prioritizing a functional endpoint rather than pain intensity alone (11, 15). The results are broadly consistent with prior work suggesting that vitamin D-related exposure or status may correlate more consistently with broader musculoskeletal function and disability than with pain severity alone (30–32), although attenuation after adjustment for sleep and depressive symptoms also indicates the likelihood of behavioral and psychosocial confounding (33–35).

Trial evidence on vitamin D supplementation for chronic pain and low back pain has been mixed, with many syntheses reporting small or null average effects across diverse populations (15, 16). Differences in baseline deficiency, achieved repletion, dose, duration, co-interventions, and pain phenotype likely contribute to this heterogeneity (36, 37). Accordingly, the present cross-sectional findings should not be read as resolving trial inconsistency, but rather as identifying a functional-outcome association that may justify more targeted prospective evaluation (38, 39).

Spine-specific literature, including work in spinal fusion and deformity pathways, has highlighted high prevalence of vitamin D deficiency and possible associations with patient-reported outcomes (19, 20). Much of that literature focuses on surgical outcomes, fusion biology, or postoperative recovery, whereas the current study emphasizes chronic outpatient symptoms across anesthesiology pain, spine surgery, and rehabilitation clinics (21). The overlap is clinically meaningful because outpatient symptom burden often influences timing of surgery, readiness for rehabilitation, and perioperative risk stratification (19). The present findings therefore complement spine surgery literature by providing evidence in the preoperative and nonoperative symptom domain, using pain interference as the primary endpoint (20).

Several mechanisms could plausibly link higher vitamin D-related exposures to lower pain interference while remaining compatible with modest effect sizes (34, 40). Vitamin D is relevant to musculoskeletal performance, neuromuscular coordination, inflammatory signaling, sleep, and mood, all of which may influence functional disability even when pain intensity changes little (41–43). Because poor sleep and depressive symptoms were highly prevalent in this cohort, and because measurement error in those domains remains possible, the observed associations may reflect overlapping biological and behavioral pathways rather than a single direct mechanism (34, 41, 43).

The stronger association with pain interference relative to pain severity is clinically and conceptually coherent (44). Pain interference integrates multiple dimensions including physical function, sleep disruption, and role participation (44, 45). If vitamin D related exposure contributes to better neuromuscular performance, improved sleep patterns, or reduced fatigue, the primary observable signal may occur in interference rather than intensity (17, 46). This pattern also supports the choice of a functional endpoint for future pragmatic trials, because functional improvement may be more meaningful to patients and more aligned with clinical decision-making in pain and spine clinics (45).

The observed effect modification by BMI and physical activity provides additional interpretive clues (47). Higher adiposity is associated with lower circulating vitamin D and altered bioavailability, and sedentary behavior can cluster with reduced sun exposure and poorer musculoskeletal conditioning (48, 49). Stronger dietary associations among participants with higher BMI and low activity may reflect a combination of biological susceptibility and behavioral clustering (50). An alternative explanation is differential measurement error: more active individuals may have higher outdoor exposure and broader diet quality, making a short dietary screener less discriminating (51). Another explanation is ceiling effects in function: higher activity groups may have lower baseline interference, leaving less room to detect exposure gradients (52). Although interaction findings should be interpreted cautiously, the pattern supports targeted hypotheses for future studies, including stratified screening strategies or trials enriched for higher-risk phenotypes (53). Residual confounding also remains likely despite broad covariate adjustment (27, 28). Although the models incorporated age, BMI, education, physical activity, sleep quality, depressive symptoms, sun exposure proxies, season, smoking, alcohol use, pain characteristics, and analgesic use pattern, other factors may influence both vitamin D-related exposure and pain outcomes (29, 54). These include socioeconomic circumstances beyond education alone, overall diet quality and nutritional patterns unrelated to vitamin D, inflammatory or autoimmune conditions, long-term corticosteroid exposure, rehabilitation intensity, health-seeking behavior, and clinician engagement (28, 55, 56). In observational nutrition-pain research, such factors can cluster strongly and are difficult to measure completely, which limits causal interpretation (28, 57).

The persistence of independent associations in mutual adjustment models suggests that dietary exposure ranking and regular supplementation capture overlapping but distinct aspects of vitamin D related behavior (58). Dietary intake is influenced by food access, preferences, and overall dietary patterns, whereas supplementation can reflect clinician advice, patient motivation, and health system contact (23). In settings where fortification is heterogeneous and dietary assessment is imperfect, supplement behavior may serve as a more stable marker of sustained intake (59, 60). The joint model results support a framing that both dietary and supplemental sources deserve consideration in clinical counseling and research design, even when precise absolute intake estimation is not feasible (61). An important measurement issue is the discrepancy between reported intake and physiological vitamin D status (27). Dietary ranking and supplement use capture exposure-related behavior, but they do not directly measure circulating 25-hydroxyvitamin D (27). Biological status depends not only on intake, but also on sun exposure, adiposity, absorption, metabolism, comorbid disease, and adherence (54, 62, 63). Accordingly, some participants may have been misclassified with respect to true physiological vitamin D status despite similar reported intake or supplement patterns (27). If such misclassification was predominantly non-differential, it would be expected to attenuate associations toward the null. However, differential misclassification is also possible if participants with higher symptom burden recalled diet, supplement use, or portion sizes differently. These considerations reinforce that the observed estimates should be interpreted cautiously and as exposure-behavior associations rather than biomarker-based effects (64).

For tertiary anesthesiology pain clinics and high-volume spine centers, including deformity and severe scoliosis pipelines, a central operational challenge is improving function while limiting harm from escalating pharmacologic exposure (65, 66). Pain interference is closely tied to disability, sleep disturbance, and psychosocial burden, and it often drives decisions about interventions, rehabilitation intensity, and surgical timing (67, 68). If vitamin D-related exposures are associated with lower interference, even modestly, the finding is clinically relevant because vitamin D assessment and counseling are comparatively low-cost, scalable, and compatible with multimodal care (23, 61). However, the present study does not establish that vitamin D optimization reduces pain interference and should not be interpreted as evidence for vitamin D as a standalone analgesic strategy. Rather, it supports further prospective evaluation within broader care models that include rehabilitation, sleep-focused management, and mood screening (69). In addition, the subgroup pattern suggests potential prioritization of patients with higher BMI or low activity for assessment and counseling, because that combination may align with greater vulnerability and higher baseline interference (70).

At the same time, clinical translation requires caution. Cross-sectional associations can reflect reverse causation, whereby individuals with lower interference are more likely to engage in healthier diet patterns, outdoor activity, and supplement adherence (71, 72). The results should therefore be viewed as hypothesis-generating and as justification for prospective designs that can better separate cause from consequence (73, 74).

### 4.1 Limitations and implications

Several limitations warrant emphasis. First, the observational cross-sectional design precludes causal inference and does not establish temporal ordering between vitamin D-related exposures and pain interference. Accordingly, reverse causation remains plausible even after multivariable adjustment. Participants with lower pain interference may have been more able to maintain healthier diets, adhere to supplement routines, spend more time outdoors, and participate in rehabilitation or other health-promoting behaviors, whereas those with greater functional interference may have had lower mobility, poorer self-care, and reduced adherence. Second, residual confounding remains likely despite adjustment for a broad set of measured covariates, including age, BMI, education, physical activity, depressive symptoms, sleep quality, sun exposure proxies, season, smoking, alcohol use, pain characteristics, and analgesic use pattern. Unmeasured or incompletely measured factors such as broader socioeconomic position, overall diet quality, inflammatory or autoimmune disease burden, long-term corticosteroid exposure, rehabilitation intensity, clinician engagement, and general health-seeking behavior may still have influenced both vitamin D-related exposures and pain outcomes. Third, dietary exposure was operationalized as an intake-ranking index rather than absolute intake, which improves feasibility in a heterogeneous fortification environment but introduces measurement error and limits direct comparability with studies reporting IU/day. The dietary screener was designed as a pragmatic focused ranking tool and was not formally validated against serum 25-hydroxyvitamin D or a comprehensive dietary reference instrument in this cohort. Fourth, supplement exposure and portion sizes relied on self-report over a 3-month recall period and are therefore vulnerable to recall bias and exposure misclassification. Finally, reported intake and supplement use do not necessarily correspond to physiological vitamin D status because status also depends on sun exposure, adiposity, absorption, metabolism, and adherence. Taken together, these considerations indicate that the observed estimates should be interpreted cautiously as adjusted associations rather than evidence of an independent causal effect.

Implications are pragmatic. Findings support routine inclusion of vitamin D related exposure assessment in observational spine pain research using function-oriented endpoints, and they justify prospective cohorts or pragmatic trials that test whether targeted vitamin D optimization, combined with rehabilitation and sleep-focused care, can improve pain interference in high-risk subgroups such as patients with higher BMI or low activity.

### 4.2 Future directions

Future work should prioritize designs that strengthen causal interpretation. A prospective cohort with repeated measures of pain interference, activity, sleep, mood, and vitamin D related exposures can clarify directionality and quantify within-person changes. Incorporation of serum 25-hydroxyvitamin D in a subset would allow calibration of intake ranking and help identify deficiency thresholds relevant to function. Pragmatic trials could enrich enrollment for patients with low activity or higher BMI, because subgroup findings suggest higher yield in that phenotype. Intervention designs should consider multimodal strategies, pairing vitamin D optimization with structured exercise or rehabilitation and sleep interventions, since disability in chronic spine-related pain is multifactorial and rarely responsive to a single lever. Finally, spine surgery pathways provide a compelling translational platform, because preoperative optimization and postoperative rehabilitation are structured touchpoints where diet and supplementation counseling can be implemented and monitored alongside functional outcomes.

## 5 Conclusion

In Chinese adults with chronic spine-related pain, higher dietary vitamin D exposure ranking and vitamin D supplementation, particularly regular supplementation, were associated with lower pain-related functional interference. These associations persisted after adjustment for a broad set of measured covariates, including physical activity, depressive symptoms, sleep quality, sun exposure proxies, season, analgesic use pattern, and pain characteristics. The observed associations were modest in magnitude and were more pronounced for pain interference than for pain severity, supporting the value of disability-oriented outcomes when evaluating nutrition-related correlates of chronic pain. Joint models suggested that dietary exposure ranking and regular supplementation captured partially distinct exposure-related information. Stronger associations among participants with higher BMI and among participants with low physical activity identify subgroups that may merit focused evaluation in future studies. Because the study was cross-sectional and subject to residual confounding, recall error, exposure misclassification, and reverse causation, the findings should be interpreted as hypothesis-generating associations rather than evidence of therapeutic benefit. Prospective cohorts and pragmatic trials are needed to determine whether vitamin D-related intake optimization is linked to improved functional outcomes when integrated with rehabilitation and sleep-focused care in tertiary spine and pain management settings.

## Data Availability

The raw data supporting the conclusions of this article will be made available by the authors, without undue reservation.
